# Autosomal Minor Histocompatibility Antigens: How Genetic Variants Create Diversity in Immune Targets

**DOI:** 10.3389/fimmu.2016.00100

**Published:** 2016-03-15

**Authors:** Marieke Griffioen, Cornelis A. M. van Bergen, J. H. Frederik Falkenburg

**Affiliations:** ^1^Department of Hematology, Leiden University Medical Center, Leiden, Netherlands

**Keywords:** allogeneic stem cell transplantation, hematological malignancy, graft-versus-leukemia reactivity, graft-versus-host disease, donor lymphocyte infusion, T-lymphocytes, minor histocompatibility antigens, immunotherapy

## Abstract

Allogeneic stem cell transplantation (alloSCT) can be a curative treatment for hematological malignancies. Unfortunately, the desired anti-tumor or graft-versus-leukemia (GvL) effect is often accompanied with undesired side effects against healthy tissues known as graft-versus-host disease (GvHD). After HLA-matched alloSCT, GvL and GvHD are both mediated by donor-derived T-cells recognizing polymorphic peptides presented by HLA surface molecules on patient cells. These polymorphic peptides or minor histocompatibility antigens (MiHA) are produced by genetic differences between patient and donor. Since polymorphic peptides may be useful targets to manipulate the balance between GvL and GvHD, the dominant repertoire of MiHA needs to be discovered. In this review, the diversity of autosomal MiHA characterized thus far as well as the various molecular mechanisms by which genetic variants create immune targets and the role of cryptic transcripts and proteins as antigen sources are described. The tissue distribution of MiHA as important factor in GvL and GvHD is considered as well as possibilities how hematopoietic MiHA can be used for immunotherapy to augment GvL after alloSCT. Although more MiHA are still needed for comprehensive understanding of the biology of GvL and GvHD and manipulation by immunotherapy, this review shows insight into the composition and kinetics of *in vivo* immune responses with respect to specificity, diversity, and frequency of specific T-cells and surface expression of HLA–peptide complexes and other (accessory) molecules on the target cell. A complex interplay between these factors and their environment ultimately determines the spectrum of clinical manifestations caused by immune responses after alloSCT.

## Allogeneic Stem Cell Transplantation

Allogeneic stem cell transplantation (alloSCT) can be a curative treatment for hematological malignancies. In alloSCT, the immune system from a healthy donor is transplanted into the patient to induce an effective response against the leukemic cells (1, 2). Unfortunately, desired anti-tumor or graft-versus-leukemia (GvL) reactivity is often accompanied with graft-versus-host disease (GvHD) affecting predominantly skin, gut, liver, and lungs (3–6). To reduce GvHD, donor T-cells can be (partially) depleted from the stem cell graft (7–9). T-cell depletion decreases the incidence and severity of GvHD, but increases the risk of leukemia relapse and opportunistic infections. Once toxicity of the conditioning has subsided, donor T-cells can be administered after alloSCT as donor lymphocyte infusions (DLI) to reinstall beneficial GvL (10, 11). Although DLI can induce long-lasting clinical remissions, GvHD remains a major cause of morbidity and mortality. Since DLI is applied to patients who do not receive (or only limited) immunosuppression, postponed DLI after alloSCT creates an ideal platform to study specificity, magnitude, and duration of GvL and GvHD.

## Minor Histocompatibility Antigens

To minimize GvHD, patients with hematological malignancies are preferably transplanted with HLA-matched donors (12, 13). After HLA-matched alloSCT, donor-derived T-cells can mediate GvL and GvHD by recognizing polymorphic peptides presented on patient cells by shared HLA molecules. In the classical dogma, intracellular proteins are degraded in the cytosol by the proteasome and peptides are presented by HLA class I to CD8 T-cells (14, 15). HLA class II molecules present peptides derived from intra- and extracellular proteins to CD4 T-cells (14, 16). In the autologous situation, peptides from normal cellular proteins cannot be recognized by the immune system due to negative selection and deletion of self-specific T-cells in the thymus (17). After HLA-matched alloSCT, however, donor T-cells recognize polymorphic peptides presented on patient cells by shared HLA as “non-self.” These polymorphic peptides or so-called minor histocompatibility antigens (MiHA) can be encoded by the male-specific Y-chromosome (H-Y antigens) or other chromosomes (autosomal MiHA) and are produced by genetic differences between patient and donor (18–21). This review is focused on discovery strategies and molecular mechanisms behind autosomal MiHA.

## T-Cell Isolation

Minor histocompatibility antigen-specific T-cells can be directly isolated from *in vivo* immune responses after alloSCT or generated *in vitro* by stimulating antigen-experienced or naive donor T-cells (22, 23). T-cells have been isolated from *in vivo* immune responses by their capacity to produce IFN-γ upon *in vitro* stimulation with patient hematopoietic cells (24, 25). A disadvantage is that not all specific T-cells produce IFN-γ during 5–24 h of incubation, resulting in low T-cell isolation efficiencies. HLA-DR is a marker that has successfully been used to isolate *in vivo* activated T-cells (26, 27). Since HLA-DR is expressed on activated T-cells for a prolonged time, its expression does not require *in vitro* stimulation and enables analysis of the *in vivo* immune response without introducing a bias. CD137 is another marker that allows direct isolation of antigen-experienced T-cells (28, 29). This marker is specific for T-cells that are recently activated and requires *in vitro* stimulation for re-expression on the cell surface.

## Discovery Strategies

To develop strategies that allow manipulation of GvL and GvHD, the dominant repertoire of autosomal MiHA needs to be discovered. HA-1 and HA-2 are the first MiHA that have been identified as T-cell targets in a patient with GvHD (Table 1). The antigens have been characterized as peptides eluted from HLA surface molecules that are recognized by specific T-cells by mass spectrometry. Other MiHA characterized by this approach are HA-8, HA-3, PANE1, and LB-ADIR-1F (Table 1). cDNA library screening in which pools of plasmids are tested for T-cell recognition is another technique that has been used for discovery of HB-1H, UGT2B17/A29, UGT2B17/B44, ACC-4, ACC-5, ACC-6, SP110, LB-ECGF-1H, C19ORF48, TRIM22, and LB-TRIP10-1EPC (Table 1). In addition, five HLA class II-restricted MiHA encoded by *PI4K2B*, *PTK2B*, *LY75*, *MR1*, and *MTHFD1* have been characterized by screening a library in which recombinant bacteria are screened for T-cell recognition (Table 1).

**Table 1:** **HLA class I- and II-restricted autosomal minor histocompatibility antigens**.

HLA-I MiHA	MiHA/AV^a^	Gene	Variant	rs number	Location	Transcript^b^	Protein^b^	HLA	Reference
HA-3	V[**T**/M]EPGTAQY	*AKAP13*	SNP	rs2061821	Exon	Normal	Normal	A*01:01	(30)
HA-2	YIGEVLVS[**V**/M]	*MYO1G*	SNP	rs61739531	Exon	Normal	Normal	A*02:01	(31)
HA-1/A2	VL[**H**/R]DDLLEA	*HMHA1*	SNP	rs1801284	Exon	Normal	Normal	A*02:01	(32)
HA-8	**[R**/P]TLDKVLEV	*KIAA0020*	SNP	rs2173904	Exon	Normal	Normal	A*02:01	(33)
LB-ADIR-1F	SVAPALAL[**F**/S]PA	*TOR3A*	SNP	rs2296377	Exon	Normal	Alternative	A*02:01	(34)
C19ORF48	CIPPD[**S**/T]LLFPA	*C19ORF48*	SNP	rs3745526	Exon	Normal	Alternative	A*02:01	(35)
TRIM22	MAVPPC[**C**/R]IGV	*TRIM22*	SNP	rs187416296	Exon	Normal	Normal	A*02:01	(22)
LB-PRCP-1D	FMWDVAE[**D**/E]LKA	*PRCP*	SNP	rs2298668	Exon	Normal	Normal	A*02:01	(27)
LB-SSR1-1S	[**S**/L]LAVAQDLT	*SSR1*	SNP	rs10004	Exon	Normal	Normal	A*02:01	(27)
LB-WNK1-1I	RTLSPE[**I**/M]ITV	*WNK1*	SNP	rs12828016	Exon	Normal	Normal	A*02:01	(27)
T4A	GLYTYWSAG[**A**/E]	*TRIM42*	SNP	rs9876490	Exon	Normal	Normal	A*02:01	(36)
UTA2-1	QL[**L**/P]NSVLTL	*KIAA1551*	SNP	rs2166807	Exon	Normal	Normal	A*02:01	(37)
LB-HIVEP1-1S	SLPKH[**S**/N]VTI	*HIVEP1*	SNP	rs2228220	Exon	Normal	Normal	A*02:01	(38)
LB-NISCH-1A	ALAPAP[**A**/V]EV	*NISCH*	SNP	rs887515	Exon	Normal	Normal	A*02:01	(38)
UGT2B17/A2	CVATMIFMI	*UGT2B1*	Gene deletion			Polymorphic	Polymorphic	A*02:06	(39)
PANE1	RVWDLPGVLK	*CENPM*	SNP	rs5758511	Exon	Alternative	Polymorphic	A*03:01	(40)
SP110	SLP[**R**/G]GTSTPK	*SP110*	SNP	rs1365776	Exon	Normal	Normal	A*03:01	(41)
ACC-1Y	DYLQ[**Y**/C]VLQI	*BCL2A1*	SNP	rs1138357	Exon	Normal	Normal	A*24:02	(42)
ACC-1C	DYLQ[Y/**C**]VLQI	*BCL2A1*	SNP	rs1138357	Exon	Normal	Normal	A*24:02	(43)
UGT2B17/A29	AELLNIPFLY	*UGT2B17*	Gene deletion			Polymorphic	Polymorphic	A*29:02	(44)
P2RX7	WFHHC[**H**/R]PKY	*P2RX7*	SNP	rs7958311	Exon	Normal	Normal	A*29:02	(45)
ACC-4	ATLPLLCA[**R**/G]	*CTSH*	SNP	rs2289702	Exon	Normal	Normal	A*31:01	(46)
ACC-5	WATLPLLCA[**R**/G]	*CTSH*	SNP	rs2289702	Exon	Normal	Normal	A*33:03	(46)
LRH-1	TPNQRQNVC	*P2X5*	INDEL	rs3215407	Exon	Normal	Polymorphic	B*07:02	(47)
LB-ECGF-1H	RP[**H**/R]AIRRPLAL	*TYMP*	SNP	rs112723255	Exon	Normal	Alternative	B*07:02	(48)
LB-APOBEC3B-1K	[**K**/E]PQYHAEMCF	*APOBEC3B*	SNP	rs2076109	Exon	Normal	Normal	B*07:02	(27)
LB-ARHGDIB-1R	LPRACW[**R**/P]EA	*ARHGDIB*	SNP	rs4703	Exon	Normal	Alternative	B*07:02	(27)
LB-BCAT2-1R	QP[**R**/T]RALLFVIL	*BCAT2*	SNP	rs11548193	Exon	Normal	Normal	B*07:02	(27)
LB-EBI3-1I	RPRARYY[**I**/V]QV	*EBI3*	SNP	rs4740	Exon	Normal	Normal	B*07:02	(27)
LB-ERAP1-1R	HPRQEQIALLA	*ERAP1*	SNP	rs26653	Exon	Normal	Normal	B*07:02	(27)
LB-GEMIN4-1V	FPALRFVE[**V**/E]	*GEMIN4*	SNP	rs4968104	Exon	Normal	Normal	B*07:02	(27)
LB-PDCD11-1F	GPDSSKT[**F**/L]LCL	*PDCD11*	SNP	rs2986014	Exon	Normal	Normal	B*07:02	(27)
ZAPHIR	IPRDSWWVEL	*ZNF419*	SNP	rs2074071	Exon	Polymorphic	Polymorphic	B*07:02	(49)
LB-FUCA2-1V	RLRQ[**V**/M]GSWL	*FUCA2*	SNP	rs3762002	Exon	Normal	Normal	B*07:02	(50)
LB-TEP1-1S	APDGAKVA[**S**/P]L	*TEP1*	SNP	rs1760904	Exon	Normal	Normal	B*07:02	(51)
HEATR1	ISKERA[**E**/G]AL	*HEATR1*	SNP	rs2275687	Exon	Normal	Normal	B*08:01	(23)
HA-1/B60	KECVL[**H**/R]DDL	*HMHA1*	SNP	rs1801284	Exon	Normal	Normal	B*40:01	(52)
LB-NUP133-1R	SEDLILC[**R**/Q]L	*NUP133*	SNP	rs1065674	Exon	Normal	Normal	B*40:01	(53)
LB-SON-1R	SETKQ[**R**/C]TVL	*SON*	SNP	rs13047599	Exon	Normal	Normal	B*40:01	(53)
LB-SWAP70-1Q	MEQLE[**Q**/E]LEL	*SWAP70*	SNP	rs415895	Exon	Normal	Normal	B*40:01	(53)
LB-TRIP10-1EPC	G[**E**/G][**P**/S]QDL[**C**/G]TL	*TRIP10*	SNP	rs1049229	3′ UTR	Normal	Alternative	B*40:01	(53)
				rs1049230					
				rs1049232					
SLC1A5	AE[**A**/P]TANGGLAL	*SLC1A5*	SNP	rs3027956	Exon	Normal	Normal	B*40:02	(39)
HB-1H	EEKRGSL[**H**/Y]VW	*HMHB1*	SNP	rs161557	Exon	Normal	Normal	B*44:03	(54)
HB-1Y	EEKRGSL[H/**Y**]VW	*HMHB1*	SNP	rs161557	Exon	Normal	Normal	B*44:03	(55)
UGT2B17/B44	AELLNIPFLY	*UGT2B17*	Gene deletion			Polymorphic	Polymorphic	B44	(44)
ACC-2	KEFED[**D**/G]IINW	*BCL2A1*	SNP	rs3826007	Exon	Normal	Normal	B*44:03	(42)
ACC-6	MEIFIEVFSHF	*HMSD*	SNP	rs9945924	Intron	Polymorphic	Polymorphic	B*44:03	(56)
DPH1	S[**V**/L]LPEVDVW	*DPH1*	SNP	rs35394823	Exon	Normal	Normal	B*57:01	(45)

**HLA-II MiHA**	**MiHA/AV^a^**	**Gene**	**Variant**	**rs number**	**Location**	**Transcript^b^**	**Protein^b^**	**HLA**	**Reference**

LB-MTHFD1-1Q	SSIIAD[**Q**/R]IALKL	*MTHFD1*	SNP	rs2236225	Exon	Normal	Normal	DRB1*03:01	(26)
LB-LY75-1K	LGITYR[**N**/K]KSLMWF	*LY75*	SNP	rs12692566	Exon	Normal	Normal	DRB1*13:01	(26)
SLC19A1	[**R**/H]LVCYLCFY	*SLC19A1*	SNP	rs1051266	Exon	Normal	Normal	DRB1*15:01	(57)
LB-PTK2B-1T	VYMND[**T**/K]SPLTPEK	*PTK2B*	SNP	rs751019	Exon	Normal	Normal	DRB3*01:01	(26)
LB-MR1-1R	YFRLGVSDPI[**R**/H]G	*MR1*	SNP	rs2236410	Exon	Normal	Normal	DRB3*02:02	(26)
CD19	WEGEPPC[**L**/V]P	*CD19*	SNP	rs2904880	Exon	Normal	Normal	DQB1*02:01	(58)
LB-PI4K2B-1S	SRSS[**S**/P]AELDRSR	*PI4K2B*	SNP	rs313549	Exon	Normal	Normal	DQB1*06:03	(59)
UTDP4-1	R[**I**/N]LAHFFCGW	*ZDHHC12*	SNP	rs11539209	Exon	Normal	Normal	DPB1*04	(60)

*^a^The polymorphic amino acid in the epitope is indicated between brackets (MiHA/AV = allelic variant)*.

*^b^Normal and alternative transcripts and proteins are expressed independently of the SNP in both patient and donor, whereas polymorphic transcripts and proteins are *de novo* created by the SNP and, thus, restricted to the patient*.

Due to advanced array techniques to measure single nucleotide polymorphisms (SNPs), whole genome association scanning (WGAs) became available as efficient method for MiHA discovery (39, 43). In this approach, a panel of test cells with known SNP genotypes is used to measure T-cell recognition. T-cell recognition is subsequently investigated for association with individual SNPs to identify the genomic region that encodes the MiHA. Before SNP arrays became commercially available, WGAs was performed with low-resolution genetic markers, leading to identification of large genomic regions of which all genes needed to be investigated for encoding the antigen. MiHA characterized by WGAs with low-resolution markers are ACC-1Y, ACC-2, LRH-1, and HEATR1 (Table 1). When high-resolution SNP data are used, WGAs enables direct identification of the MiHA-producing SNP or identification of small genomic regions with SNP(s) that are in linkage disequilibrium with the MiHA-producing SNP. MiHA identified with high-­resolution SNP data are ACC-1C, SLC1A5, UGT2B17/A2, DPH1, P2RX7, LB-PRCP-1D, SSR1-1S, LB-WNK1-1I, LB-EBI3-1I, LB-BCAT2-1R, LB-ARHGDIB-1R, LB-PDCD11-1F, LB-APOBEC3B-1K, LB-GEMIN4-1V, LB-ERAP1-1R, ZAPHIR, LB-SON-1R, LB-NUP133-1R, LB-SWAP70-1Q, UTA2-1, and LB-FUCA2-1V (Table 1). WGAs with high-resolution SNPs also led to discovery of HLA class II-restricted MiHA encoded by *CD19*, *SLC19A1*, and *ZDHHC12* (Table 1). Nowadays, data for all SNPs as present in the human genome are available in the 1000 Genomes Project and the value of this dataset has recently been illustrated by discovery of UTDP4-1 (Table 1).

Whereas T-cells are used to identify MiHA by forward strategies; in reverse strategies, peptides are selected to search for specific T-cells. Polymorphic peptides identified in HLA-ligandomes (38, 51), hematopoiesis-restricted genes (52, 55, 61), and peptides identified based on association of SNPs with good clinical outcome after alloSCT (36) have been selected to search for specific T-cells in transplanted patients or healthy individuals.

In total, 48 HLA class I-restricted and 8 HLA class II-restricted autosomal MiHA have thus far been characterized. These numbers are expected to rapidly increase in the near future, in particular, if WGAs is performed with cell panels for which all SNPs are measured by whole genome sequencing.

## Molecular Mechanisms

**Figure 1:**
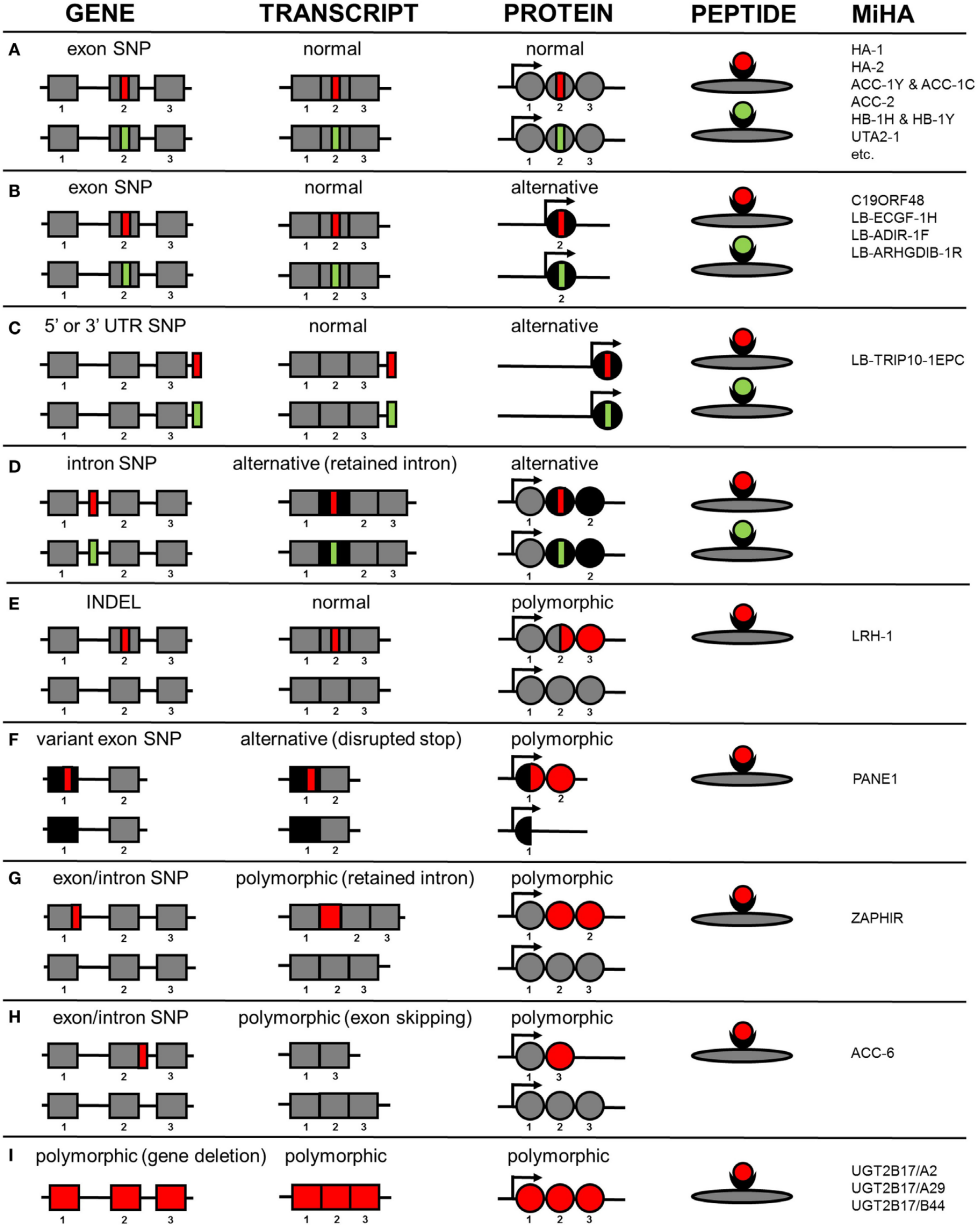
**Molecular mechanisms by which genetic variants create autosomal MiHA**. Normal non-polymorphic sequences are indicated in gray, whereas alternative non-polymorphic sequences are shown in black. Polymorphic patient-specific sequences are shown in red and donor-specific sequences are indicated in green (if allelic variants exist). Whether the allelic variants are actually presented on the cell surface is also dependent on intracellular processing and presentation mechanisms, which are not taken into consideration in this figure. **(A)** MiHA created by SNPs in primary gene transcripts in the normal reading frame. **(B)** MiHA created by SNPs in primary gene transcripts in an alternative reading frame. **(C)** MiHA created by SNPs in 5´ or 3´ UTR of primary gene transcripts. **(D)** MiHA created by intron SNPs as retained in alternative gene transcripts. **(E)** MiHA derived from polymorphic proteins as created by frameshift insertions or deletions in primary gene transcripts. **(F)** MiHA derived from polymorphic proteins as created by SNP in alternative gene transcripts. **(G)** MiHA translated from intron sequences in polymorphic gene transcripts as created by exon or intron SNPs. **(H)** MiHA translated from polymorphic gene transcripts in which exon sequences are skipped as created by exon or intron SNPs. **(I)** MiHA encoded by polymorphic genes.

HLA class I-restricted autosomal MiHA are generated by different molecular mechanisms. An overview of the various mechanisms by which genetic variants create MiHA is shown in Figure 1. Of the 48 HLA class I-restricted MiHA, 36 antigens are encoded by SNPs in coding exons, leading to single amino acid changes in proteins that are translated from primary gene transcripts in the normal reading frame (Figure 1A). MiHA can, however, also be translated from normal gene transcripts in an alternative reading frame. These SNPs can be located in coding exons (C19ORF48, LB-ECGF1-1H, LB-ADIR-1F, LB-ARHGDIB-1R) (Figure 1B) or in 5’ or 3’ UTR regions (LB-TRIP10-1EPC) (Figure 1C). Though not yet discovered, it is expected that MiHA can also be encoded by SNP in intron regions that are retained in alternative transcripts (Figure 1D). Proteins translated in alternative reading frames are considered as aberrant proteins that lack any cellular function, the so-called defective ribosomal products (DRiPs). DRiPs are rapidly degraded during or shortly after translation and evidence has been found that they may be a main source of peptide precursors for T-cell immunosurveillance (62). Degradation is an important factor for HLA presentation (63), but relative abundance of normal functional proteins in the cell may counteract the actual contribution of DRiPs to the HLA-ligandome.

In addition to the proteins described above that are expressed independently of the SNP in both patient and donor, MiHA can also be derived from proteins or protein products that are *de novo* created by SNPs. Expression of these polymorphic proteins is restricted to the patient and allelic variants in the donor do not exist. As a result, the epitope that is recognized by the T-cell can be derived from another protein region than the amino acids that are directly encoded by the SNP. Examples of antigens from polymorphic proteins that are *de novo* created are LRH-1, an antigen that is produced by an insertion/deletion variant (INDEL) that induces a frameshift in protein translation (Figure 1E) and PANE1, which is an antigen from an elongated protein created by a SNP that disrupts the stop codon (Figure 1F). PANE1 and LRH-1 are both polymorphic proteins translated from transcripts that are expressed independently of the SNP in both patient and donor. However, MiHA can also be encoded by transcripts that are newly created by the SNP. These polymorphic transcripts are expressed in the patient and do not exist in the donor. Antigens encoded by polymorphic transcripts that are newly created by SNPs are ZAPHIR, which is translated from a ZNF419 transcript in which an intron is retained (Figure 1G), and ACC-6, which is encoded by an HMSD transcript that is generated by exon skipping (Figure 1H). Finally, MiHA can be encoded by polymorphic genes as illustrated by *UGT2B17*, which is present in the patient but absent in the donor genome (Figure 1I).

The numbers of MiHA that have been characterized for each molecular mechanism as shown in Figure 1 probably do not reflect the actual contribution of the various mechanisms to the entire repertoire of MiHA that are recognized by specific T-cells after alloSCT. This is suggested by the finding that for various T-cell clones, associating SNPs have successfully been identified by WGAs in genomic regions outside known exons, whereas epitope discovery failed due to absence of SNP disparities in the normal gene transcript. MiHA recognized by these T-cells are probably encoded by cryptic transcripts. RNA-sequence data can be used to search for these cryptic transcripts in the genomic region that contains the associating SNPs and single RNA-sequence reads can be analyzed to determine the exact sequence composition of the transcripts, thereby facilitating discovery of these MiHA. As such, implementation of RNA-sequence analysis in a combined approach of whole genome and transcriptome analysis may increase the efficiency of MiHA discovery. The various molecular mechanisms how genetic variants create MiHA as shown in Figure 1 are probably similar for neoantigens. Neoantigens are peptides created by tumor-specific mutations that are presented by HLA and recognized by specific T-cells (64). In cancer neoantigen discovery, research is focused on selecting peptides encoded by mutations in coding exons with single amino acid changes in the normal protein reading frame (Figure 1A), whereas other molecular mechanisms (Figures 1B–H) are often not taken into consideration. RNA-sequence analysis may, therefore, also be relevant to elucidate transcript variants for neoantigens in particular since splicing defects often occur in cancer (65).

## Tissue Distribution

The tissue distribution of MiHA is an important factor in clinical manifestations caused by immune responses after alloSCT. Various T-cells recognize leukemic cells *in vitro* with no or minimal reactivity against non-hematopoietic cells. These T-cells are expected to mediate beneficial GvL after alloSCT without GvHD. Other T-cells are reactive with both hematopoietic and non-hematopoietic cells, suggesting a role in GvHD. Since in alloSCT, patient hematopoiesis is replaced by a blood-forming system from a healthy donor, donor T-cells for hematopoiesis-restricted MiHA eliminate the malignant cells of the patient, while sparing healthy hematopoietic cells of donor origin. Therefore, discovery of hematopoietic MiHA is an explicit research goal.

Various methods are available to investigate the tissue distribution of MiHA to estimate their efficacy and toxicity as T-cell targets. Toxicity can be analyzed by measuring T-cell reactivity against non-hematopoietic cells from organs that are targeted in GvHD. This analysis, however, requires collection of a variety of tissues expressing the relevant MiHA and HLA restriction allele. Skin fibroblasts are frequently used to estimate toxicity and have also been cultured with cytokines to mimic the inflammatory environment of the early post-transplantation period. T-cells often recognize skin fibroblasts when cultured under inflammatory conditions, what may be explained by efficient antigen processing and presentation and enhanced surface expression of HLA, costimulatory, and adhesion molecules. Other non-hematopoietic cells, however, are more difficult to culture and often not available in quantities that allow in depth T-cell analysis. Therefore, as second best option, the tissue distribution can be investigated by gene expression analysis. Thus far, only a limited number of MiHA are encoded by genes with restricted or predominant expression in (malignant) hematopoietic cells, i.e., *HMHB1* (54), *MYO1G* (66), *HMHA1* (67), *BCL2A1* (42), *P2*×*5* (47), *CENPM* (40), *HMSD* (56), *KIAA1551* (37), and *ARHGDIB* (68). Although gene expression analysis allows rapid selection of hematopoietic antigens, the therapeutic value of MiHA needs to be validated by demonstrating the capacity of specific T-cells to kill leukemic cells and confirming their failure to react with non-hematopoietic cells.

## *In Vivo* Immune Responses

Minor histocompatibility antigens characterization enabled *ex vivo* quantification of specific T-cells by pMHC multimers in individual patients after alloSCT. Staining with pMHC multimers demonstrated a peak in the immune response in patients who responded to DLI after HLA-matched alloSCT (27, 37, 47, 69). In these patients, high frequencies of circulating T-cells coincided with development of GvL. Detailed analysis of peak responses between 4 and 12 weeks after DLI demonstrated that a diversity of HLA class I- and II-restricted MiHA are targeted by CD8 and CD4 T-cells (26, 27, 53). These T-cells expand and retract with similar kinetics, although frequencies and timing of the peak may differ between MiHA (27, 53).

Although GvL after alloSCT is often accompanied with GvHD, strong anti-tumor responses without severe side effects are occasionally observed (69), illustrating that GvL can be separated from GvHD. In the pathophysiology of GvHD, the tissue distribution of MiHA is important as well as the frequencies of circulating T-cells, their homing behavior and capacity to destroy non-hematopoietic cells *in situ* (70). Although tissue distribution is relevant, occurrence of GvHD cannot entirely be explained by induction of T-cells targeting MiHA on non-hematopoietic tissues. This became clear when T-cells for hematopoietic and ubiquitous MiHA were simultaneously detected in patients with severe GvHD (71) and patients without GvHD (53). Since immune responses in patients with GvHD are generally strong, it can be speculated that T-cell reactivity against non-hematopoietic tissues needs to exceed a certain threshold in GvHD (72). Since T-cells for ubiquitous MiHA may stimulate development of GvL by releasing cytokines, strategies that retain reactivity against healthy tissues below the threshold may effectively separate GvL from GvHD.

## Therapeutic Use

As the number of characterized MiHA increases, T-cells from different patients more often recognize MiHA that are already known, suggesting that the repertoire of MiHA that are presented by HLA and recognized by specific T-cells is limited and follow rules for immunodominance that cannot be predicted by measuring only SNP disparities (73). If true, a large proportion of all MiHA with balanced population frequencies will be characterized in the coming years. Discovery of these MiHA is needed to analyze and compare *in vivo* immune responses in GvL and GvHD with respect to specificity, diversity, frequency, and dynamics of specific T-cells. Moreover, it enables to follow GvL and GvHD in large patient groups, which is essential to investigate and compare efficacy and toxicity of different alloSCT (and DLI) transplantation protocols.

With the discovery of a large proportion of common MiHA, a variety of targets become available for therapy to augment GvL after alloSCT (74, 75). One strategy is *in vitro* production and adoptive transfer of donor T-cells for hematopoietic MiHA (75, 76). Patients with leukemia who relapsed after alloSCT have been treated with *in vitro* expanded T-cells for leukemic cells (77, 78), T-cells for HA-1 (79) or MiHA-specific T-cells that lacked reactivity against fibroblasts (45). Other strategies for adoptive transfer are isolation of MiHA-specific T-cells from the DLI by pMHC multimers and T-cell receptor (TCR) gene transfer. In the latter study, patients are treated with virus-specific donor T-cells that are genetically engineered with the TCR for HA-1 (80). Besides adoptive transfer, patients with hematological malignancies can be *in vivo* vaccinated with donor (or patient) dendritic cells loaded with peptides or mRNA (81–83). In conclusion, hematopoietic MiHA (and their specific TCRs) may be easily implemented in ongoing clinical trials to increase efficacy, reduce toxicity, and broaden applicability of immunotherapy after alloSCT.

## Concluding Remarks

In this review, the various molecular mechanisms how genetic variants create autosomal MiHA are described as well as the relevance of these antigens as tools to understand the biology of GvL and GvHD and as targets for immunotherapy to treat hematological cancers after alloSCT. Although more MiHA are needed for comprehensive understanding and manipulation by immunotherapy, this review shows insight into the composition and kinetics of *in vivo* immune responses with respect to specificity, diversity, and frequency of specific T-cells and surface expression of HLA-peptide complexes and other (accessory) molecules on the target cell. A complex interplay between these factors and their environment (84) ultimately determines the spectrum of clinical manifestations that are caused by immune responses after alloSCT.

## Author Contributions

All authors listed, have made substantial, direct, and intellectual contribution to the work, and approved it for publication.

## Conflict of Interest Statement

The authors declare that the research was conducted in the absence of any commercial or financial relationships that could be construed as a potential conflict of interest.

## Abbreviations

alloSCT, allogeneic stem cell transplantation; DLI, donor lymphocyte infusion; DRiP, defective ribosomal product; GvHD, graft-versus-host disease; GvL, graft-versus-leukemia reactivity; MiHA, minor histocompatibility antigen; SNP, single nucleotide polymorphism; WGAs, whole genome association scanning.
